# Correlation of Long Non-coding RNA LncRNA-FA2H-2 With Inflammatory Markers in the Peripheral Blood of Patients With Coronary Heart Disease

**DOI:** 10.3389/fcvm.2021.682959

**Published:** 2021-06-21

**Authors:** Fengxia Guo, Yanhua Sha, Bing Hu, Gang Li

**Affiliations:** ^1^Department of Clinical Laboratory, Henan Provincial People's Hospital, People's Hospital of Zhengzhou University, Zhengzhou, China; ^2^Department of Laboratory Medicine, The Second Affiliated Hospital of Guangzhou University of Chinese Medicine, Guangzhou, China; ^3^Department of Clinical Laboratory, Affiliated Cancer Hospital of Zhengzhou University, Zhengzhou, China

**Keywords:** coronary heart disease, long non-coding RNA, inflammatory marker, LncRNA-FA2H-2, peripheral blood

## Abstract

**Objective:** To characterize the expression of long non-coding RNA LncRNA-FA2H-2 in coronary heart disease (CHD) and its correlation with inflammatory markers.

**Methods:** From December 2018 to December 2020, 316 patients at Henan Provincial People's Hospital who complained of chest tightness or chest pain and had coronary angiography to clarify their coronary artery conditions for definitive diagnoses were selected as the study subjects. Plasma was collected to detect white blood cells (WBCs), total cholesterol (TG), triglyceride cholesterol (TC), low-density lipoprotein cholesterol (LDL-C), high-density lipoprotein cholesterol (HDL-C), apolipoprotein A1 (ApoA1), and C-reactive protein (CRP) levels. Tumor necrosis factor (TNF-α), monocyte chemotactic protein 1 (MCP-1), vascular cell adhesion molecule-1 (VCAM-1), intercellular cell adhesion molecule-1 (ICAM-1), and interleukin-6 (IL-6) levels were also measured using ELISA. The expression levels of lncRNA-FA2H-2 were measured using quantitative real-time PCR. The data obtained were analyzed by independent sample *t*-tests, rank sum tests, regression analyses, Pearson's or Spearman's correlation analyses, and receiver operating characteristic curves.

**Results:** (1) Compared with the control group, the differences in age, sex, diabetes, smoking, drinking, body mass index (BMI), WBC, TC, and LDL-C in CHD were not statistically significant, while the differences in hypertension, TG, HDL-C, ApoA1, and CRP were statistically significant. (2) In the grouping of coronary lesion branches, patients with age, sex, hypertension, diabetes, smoking, drinking, BMI, WBC, TC, LDL-C, HDL-C, and ApoA1 differences were not statistically significant, but TG and CRP differences were statistically significant. (3) The relative expressions of TNF-α, MCP-1, VCAM-1, ICAM-1, and IL-6 were significantly upregulated in the CHD group (*P* < 0.001). (4) The results showed that the relative levels of TNF-α, MCP-1, VCAM-1, ICAM-1, and IL-6 between the two comparative analyses (high risk, moderate risk, and low risk groups) were statistically significant. In addition, positive correlations were found between the Gensini score and TNF-α, MCP-1, VCAM-1, ICAM-1, and IL-6 in CHD patients. (5) LncRNA-FA2H-2 relative expression in the CHD group was significantly downregulated (*P* < 0.001). (6) The differences in the expression levels of LncRNA-FA2H-2 were statistically significant between the two comparative analyses (*P* < 0.01), except between the 2-branch lesion and 3-branch lesion groups. (7) LncRNA-FA2H-2 was not associated with age, sex, hypertension, diabetes, smoking, drinking, BMI, WBC, TG, TC, LDL-C, HDL-C, and ApoA1 (*P* > 0.05). (8) A correlation was found between LncRNA-FA2H-2 and MCP-1, and VCAM-1, ICAM-1, IL-6, and Gensini. (9) The results indicated that the relative levels of LncRNA-FA2H-2 between the two comparative analyses (high risk, moderate risk, and low risk groups) were statistically significant. A negative correlation was found between the Gensini score and LncRNA-FA2H-2. (10) ROC curve analyses of TNF-α, MCP-1, VCAM-1, ICAM-1, and IL-6 in CHD showed the area under the curve (AUC) = 0.832 (0.77, 0.893) with a cut-off value of 290.5, a sensitivity of 73%, and a specificity of 64%; AUC = 0.731 (0.653, 0.809) with a cut-off value of 396 and with a sensitivity of 59% and specificity of 79%; AUC = 0.822 (0.757, 0.887) with a cut-off value of 264 and with a sensitivity of 72% and specificity of 83%; AUC = 0.794 (0.715, 0.874) with a cut-off value of 201.5 and with a sensitivity of 75% and specificity of 65%; AUC = 0.760 (0.685, 0.834) with a cut-off value of 328 and with a sensitivity of 55% and specificity of 90%. (11) ROC curve analysis of LncRNA-FA2H-2 in CHD patients showed AUC = 0.834 (0.688, 0.85) with a cut-off value of 3.155 and with a sensitivity of 85% and specificity of 82%. (12) Logistic analyses showed that TNF-α, MCP-1, VCAM-1, IL-6, and LncRNA-FA2H-2 were independent risk factors for CHD.

**Conclusions:** The expression of LncRNA-FA2H-2 was reduced and inversely correlated with inflammation-related factors in CHD patients. LncRNA-FA2H-2 may have potential as an inflammatory marker for risk assessment of CHD development.

## Introduction

It is well-known that coronary heart disease (CHD) is a prevalent cardiovascular disorder (1, 2) and is the main cause of cardiovascular diseases, while the inflammatory response is present throughout the course of CHD (3–5). Coronary angiography is currently the main method of diagnosing CHD, but the tests are expensive and invasive, and unsuitable for dynamic monitoring. However, blood biochemical tests provide an important laboratory basis for the diagnosis and treatment of heart disease, especially CHD. Previous studies have shown that the levels of inflammatory biomarkers can assess the occurrence and severity of CHD (6–8), but these inflammatory indicators are susceptible to interference by the body's environment and external factors, and are therefore, unstable as indicators. An in-depth investigation of potential biomarkers of CHD could therefore be an important guide to assessing the risk of CHD.

Most (90%) of the transcribable genes in the human genome are non-coding RNAs (ncRNAs), and only 2% are protein-coding genes (9). Current research on specific long non-coding RNAs (lncRNAs) has mainly focused on tumor diseases, but the role of lncRNAs in cardiovascular diseases and its related mechanisms are not yet known. As functional studies of lncRNAs have increased, investigators have found that some lncRNAs can be used as potential target genes and biomarkers for the prevention and treatment of CHD. The current studies of LncRNAs related to CHD mainly focus on RNCR3, UCA1, LncRNA-p21, HOTAIR, ANRIL, and TUG1 (10–16). The lncRNA, RNA-ANRIL (CDKN2B-AS), localized on human chromosome 3, was found to be strongly correlated with the risk of CHD (17). In addition, LncRNA CARMEN and LncRNA fendrr are associated with embryonic heart development (18, 19). LncRNA-Ang362 may be associated with prognosis after coronary stenting (percutaneous coronary intervention) for CHD (20). Increased expression of LncRNA GAS5 was found in plaques of atherosclerotic individuals, and the mechanism may be related to exosome regulation of macrophage and endothelial cell apoptosis in atherosclerotic plaques (21). LncRNA MALAT1 was positively correlated with the expression of inflammatory mediators, IL-6 and TNF, and the mechanism may be related to the inflammatory ligand, serum amyloid 3, in endothelial cells (22). Research on LncRNAs and CHD is in the developing stage, and further studies are needed before it can be clinically used. LncRNA-FA2H-2 is a newly identified inflammation-related LncRNA reported in our previous study, and it attenuated the development of atherosclerosis *via* inhibition of the inflammatory response (23), but its expression in CHD patients is still unclear. In the present study, we characterized the expression of LncRNA-FA2H-2 in CHD patients, as well as its correlation with inflammatory markers, and we determined whether LncRNA-FA2H-2 could be used as a biomarker for predicting the risk of CHD.

## Research Subjects and Methods

### Study Subjects

This study examined patients admitted to our hospital with chest tightness or chest pain as the main complaint for definitive diagnosis between December 2018 and December 2020. The patients underwent a series of clinical examinations, and coronary angiography was finally conducted to clarify the condition of their coronary arteries. These patients were selected for the study subjects, and were divided into the control and CHD groups. The inclusion criteria for the CHD group included patients with at least one artery (left main coronary artery trunk, left anterior descending branch, left circumflex branch, and right coronary artery) having more than 50% stenosis. Inclusion criteria for the control group included coronary angiography showing no stenosis or only myocardial bridge changes (congenital anomalies of coronary artery development, which refers to a segment of the coronary artery that travels through the myocardium and is covered by a bundle of myocardial fibers called a myocardial bridge) in the coronary artery. Exclusion criteria included previous coronary surgery, including angiography and bypass surgery, active bleeding from various causes, with severe liver and kidney insufficiency, peripheral vascular disease, tumors, hematological diseases, acute and chronic infectious diseases, chronic obstructive pulmonary disease, cardiac arrhythmias, and diabetes mellitus. The study was approved by the hospital ethics committee, and each patient signed an informed consent form.

### Specimen Collection and Processing

Blood samples were taken before the administration of any drugs to the patients. We drew 7 ml of fasting venous blood from the patients in the morning and placed the samples in EDTA tubes in a temperature-controlled centrifuge. The samples were centrifuged at 3,000 rpm for 15 min. The plasma was retained for subsequent studies, and the remaining samples were stored at −80°C for cryopreservation. The expressions of WBC, TG, TC, LDL-C, HDL-C, ApoA1, and CRP in the plasma were subsequently measured.

## Methods

### ELISA

The plasma from patients was collected, and the levels of TNF-α (ab183218), MCP-1 (ab179886), VCAM-1 (ab134047), ICAM-1 (ab174445), and IL-6 (ab178013) were measured by ELISA kits purchased from Abcam (Cambridge, MA, USA). All experimental procedures were performed in accordance with the manufacturer's instructions. The absorbance values were measured using a microplate reader (Molecular Devices, San Jose, CA, USA) at a wavelength of 450 nm.

### The qRT-PCT

Total RNA was isolated from plasma using a TRIzol kit (Invitrogen, Carlsbad, CA, USA). The RNA concentration was measured with a spectrophotometer (NanoDrop® 2000; Thermo Fisher Scientific, Waltham, MA, USA). First strand complementary DNA (cDNA) of lncRNA-FA2H-2 was synthesized using M-MLV reverse transcriptase (Promega, Madison, WI, USA), while total mRNA was synthesized into cDNA using a High Capacity cDNA Reverse Transcription Kit (Thermo Fisher Scientific), both in a reaction volume of 20 μl containing 1 μg of total RNA as the template strand. The expression levels of lncRNA-FA2H-2 and U6 as endogenous controls were evaluated by RT-qPCR using the SYBR Green Master Mix (TaKaRa Bio, Beijing, China) on a LightCycler 480 Fast Real-Time PCR system (Roche Molecular Systems, Pleasanton, CA, USA). PCR reactions were run in a 10 μl final volume containing 100 ng cDNA, 0.8 μl forward and reverse primers, 5 μl SYBR-Green, and 3.2 μl ddH2O. Ploidy differences in expression levels were determined using the 2^−ΔΔCt^ method. All experimental procedures were performed according to the manufacturer's instructions. All samples were measured in triplicate and the mean values were used for comparative analysis. All primers used for RT-PCR were synthesized by Sangon Biotech (Shanghai, China). The RT-qPCR thermal cycling procedure consisted of an initial pre-incubation step of 20 s at 95°C followed by 40 cycles of 10 s at 95°C, 20 s at 60°C, and 1 s at 70°C. The U6 primer sequences were as follows: forward, GTGGCCGAGGACTTTGATTG, reverse, CCTGTAACAACGCATCTCATATT; LncRNA-FA2H-2: forward, TTCCCTTACTCAGTGGTTCCC, reverse, GCTTTCTCCAATCCTACC.

### Gensini Score

Two or more experienced investigators quantitatively scored the degree of stenosis in the four vessels of the left main coronary artery trunk, left circumflex branch, right coronary artery, and left anterior descending branch based on the imaging results using the current internationally accepted method of visual diameter measurements. The score representing the degree of coronary lesion in each patient was the sum of the scores of each branch.

**Table:** 

**Narrowness**	**Score**	**Lesion**	**Score**
1–25%	1	Left main stem	5
26–50%	2	Left anterior descending branch or left gyral branch	2.5
51–75%	4	Middle left anterior descending branch	1.5
76–90%	8	Distal segment of the left anterior descending branch	1.0
91–99%	16	Middle and distal left gyral branch	1.0
Fully closed	32	Right coronary artery	1
		Small branch	0.5

## Statistical Methods

Statistical analysis was performed using SPSS statistical software for Windows, version 23.0 (SPSS, Chicago, IL, USA). The Kolmogorov–Smirnov test was used for normal distributions, and conformity to a normal distribution was expressed as the mean ± standard deviation. The independent samples *t*-test was used for comparisons between two groups for measurement data, and one-way ANOVA was used for comparisons between multiple groups. Failure to conform to a normal distribution was expressed as the median percentile.

Comparisons between two groups were performed using the Mann–Whitney U rank sum test, and multiple comparisons were conducted using the Kruskal–Wallis rank sum test. Pearson's or Spearman's correlation analyses, ROC curves, and logistic regression analyses of risk factors for CHD patients were also used. A value of *P* < 0.05 indicated a statistically significant difference.

## Results

### Analysis of Clinical Data

The differences in age, sex, diabetes, smoking, drinking, BMI, WBC, TC, and LDL-C in the CHD group were not statistically significant (*P* > 0.05), but the differences in hypertension, TG, HDL-C, ApoA1, and CRP levels were statistically significant (*P* < 0.001) (Table 1).

**Table 1:** Comparison of clinical data.

	**Control (*n* = 100)**	**CHD (*n* = 216)**	***P***
Age (year)	55.8 ± 4.4	56.1 ± 4.1	0.669
Sex	28/22	57/49	0.54
Hypertension [case (%)]	29 (29%)	105 (48.6%)	0.001
Diabetes [case (%)]	35 (35%)	89 (41.2%)	0.089
Smoking [case (%)]	34 (34%)	94 (43.5%)	0.102
Drinking [case (%)]	40 (40%)	99 (45.8%)	0.092
BMI (Kg/m^2^)	22.18 ± 2.32	23.21 ± 2.45	0.165
WBC (10^9^)	5.23 ± 1.83	5.39 ± 1.75	0.318
TG (mmol/L)	3.59 ± 0.83	4.90 ± 0.82	0.001
TC (mmol/L)	0.85 ± 0.38	0.84 ± 0.38	0.9
LDL-C (mmol/L)	2.42 ± 0.43	2.38 ± 0.47	0.619
HDL-C (mmol/L)	1.71 ± 0.71	1.07 ± 0.2	0.001
ApoA1 (g/L)	1.32 ± 0.29	1.0 ± 0.25	0.001
CRP (mg/dL)	4.07 ± 2.32	21.66 ± 5.86	0.001

### Analysis of Clinical Data in the Grouping of the Number of Coronary Lesions

CHD patients were grouped according to the number of coronary lesion branches. There were 72 patients in the 1-branch lesion group, 80 patients in the 2-branch lesion group, and 64 patients in the 3-branch lesion group. The differences in age, sex, hypertension, diabetes, smoking, drinking, BMI, WBC, TC, LDL-C, HDL-C, and ApoA1 were not statistically significant in the three groups, but the differences in TG and CRP were statistically significant (*P* < 0.001) (Table 2).

**Table 2:** Analysis of clinical data in the grouping of the number of branches of coronary lesions.

	**1 Sticks (*n* = 72)**	**2 Sticks (*n* = 80)**	**3 Sticks (*n* = 64)**	***P***
Age (year)	52.1 ± 3.8	54.8 ± 4.2	53.5 ± 4.4	0.589
Sex (male/female)	37/35	42/38	31/33	0.47
Hypertension [case (%)]	32 (44.4%)	38 (47.5%)	35 (54.6%)	0.352
Diabetes [case (%)]	25 (34.7%)	34 (42.5%)	30 (46.8%)	0.673
Smoking [case (%)]	30 (41.6%)	35 (43.7%)	29 (45.3%)	0.701
Drinking [case (%)]	38 (52.7%)	33 (41.2%)	28 (43.7%)	0.328
BMI (Kg/m^2^)	23.34 ± 2.78	23.09 ± 2.18	22.10 ± 2.31	0.173
WBC (10^9^)	4.9 ± 1.67	5.2 ± 1.85	5.01 ± 1.54	0.207
TG (mmol/L)	3.96 ± 0.45	4.34 ± 1.00	5.23 ± 0.83	0.001
TC (mmol/L)	0.81 ± 0.42	0.83 ± 0.57	0.81 ± 0.33	0.458
LDL-C (mmol/L)	2.34 ± 0.23	2.41 ± 0.32	2.30 ± 0.46	0.420
HDL-C (mmol/L)	1.65 ± 0.39	1.74 ± 0.43	1.68 ± 0.54	0.314
ApoA1 (g/L)	1.29 ± 0.17	1.21 ± 0.28	1.26 ± 0.32	0.183
CRP (mg/dL)	10.23 ± 1.30	19.34 ± 3.29	23.29 ± 1.7	0.001

### Analyses of Expression Levels of TNF-α, MCP-1, VCAM-1, ICAM-1, and IL-6 in CHD Patients

We analyzed the expression levels of TNF-α, MCP-1, VCAM-1, ICAM-1, and IL-6 using ELISA kits. The expression levels of TNF-α, MCP-1, VCAM-1, ICAM-1, and IL-6 were significantly higher in CHD patients (all, *P* < *0.001*) (Table 3).

**Table 3:** Comparison of inflammation-related factors.

	**Control (*n* = 100) P50 (P25, P75)**	**CHD (*n* = 216) P50 (P25, P75)**	***P***
TNF-α (pg/ml)	213 (95.75, 276)	393 (257.75, 432)	<0.001
MCP-1 (pg/ml)	327 (120.25, 360)	418.5 (271.75, 538.75)	<0.001
VCAM-1 (pg/ml)	172.5 (108, 192)	320 (230, 384.75)	<0.001
ICAM-1 (pg/ml)	138 (109, 183)	294 (196.5, 322.5)	<0.001
IL-6 (pg/ml)	193.5 (110.5, 285.25)	354 (202.25, 467)	<0.001

### Correlation Analysis Between the Gensini Score and TNF-α, MCP-1, VCAM-1, ICAM-1, and IL-6 in CHD Patients

**Figure 1:**
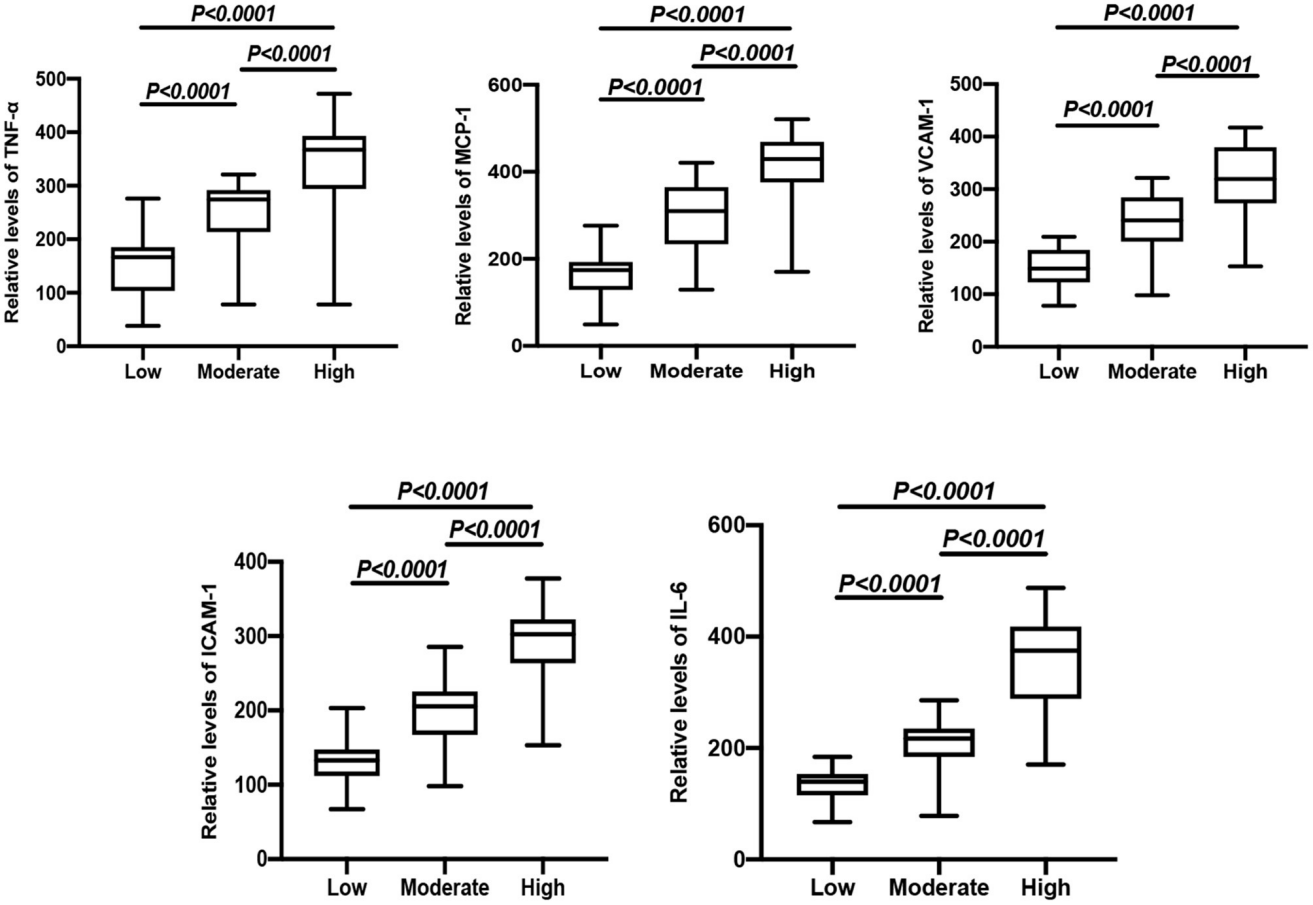
Comparison of relative expressions of TNF-α, MCP-1, VCAM-1, ICAM-1, and IL-6 in the high risk, moderate risk, and low risk groups.

CHD patients were grouped into high risk, moderate risk, and low risk groups according to their Gensini score. The results showed that the relative levels of TNF-α, MCP-1, VCAM-1, ICAM-1, and IL-6 between the two comparative analyses were statistically significant (Figure 1). In addition, positive correlations were found between the Gensini score and TNF-α, MCP-1, VCAM-1, ICAM-1, and IL-6 in CHD patients (Table 4).

**Table 4:** Correlation analysis of the Gensini score and TNF-α, MCP-1, VCAM-1, ICAM-1, and IL-6.

	**Gensini score**	
	**R**	***P***
TNF-α	0.231	0.045
MCP-1	0.218	0.032
VCAM-1	0.173	0.029
ICAM-1	0.157	0.020
IL-6	0.225	0.013

### Analysis of the Expression Level of LncRNA-FA2H-2 in CHD Patients

**Figure 2:**
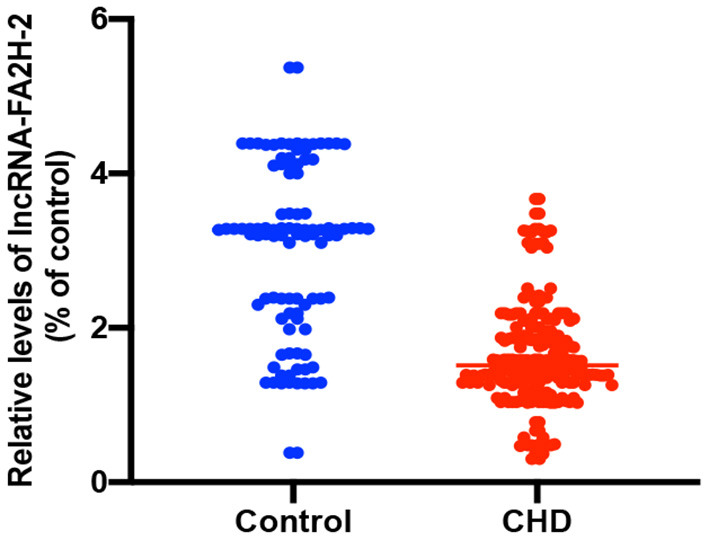
Analysis the relative expression of LncRNA-FA2H-2.

The relative expression of LncRNA-FA2H-2 was significantly downregulated in CHD patients when compared with the control group (*P* < 0.001) (Table 5 and Figure 2).

**Table 5:** Analysis the relative expression of LncRNA-FA2H-2.

	**Control (*n* = 100) P50 (P25, P75)**	**CHD (*n* = 216) P50 (P25, P75)**	***P***
LncRNA-FA2H-2	3.24 (2.17, 4.1)	1.57 (1.26, 2.46)	<0.001

### Detection of the Expression Level of LncRNA-FA2H-2 in Lesion Branch Groupings

CHD patients were grouped according to the number of coronary lesion branches, with 72 patients in the 1-branch lesion group, 80 patients in the 2-branch lesion group, and 64 patients in the 3-branch lesion group, while the control group was defined as the 0-branch lesion group. As shown in Table 6 below, the differences between the two comparative analyses were statistically significant (*P* < 0.01), except for no statistical difference between the 2-branch lesion and 3-branch lesion groups.

**Table 6:** The expression levels of LncRNA-FA2H-2 in lesion branch number subgroups.

**Number of sticks**	**LncRNA-FA2H-2**
0	3.24 (2.17, 4.1)^bcd^
1	1.98 (1.59, 2.13)^acd^
2	1.59 (1.26, 1.89)^ab^
3	1.27 (1.26, 2.34)^ab^

*For a compared with the 0-branch lesion group, P < 0.01 was statistically significant; for b compared with the 1-branch lesion group, P < 0.01 was statistically significant; for c compared with the 2-branch lesion group, P < 0.01 was statistically significant; and for d compared with the 3-branch lesion group, P < 0.01 was statistically significant*.

### Correlation Analysis of LncRNA-FA2H-2 With Clinical Characteristics in CHD Patients

LncRNA-FA2H-2 showed no correlation (*P* > 0.05) with age, sex, hypertension, diabetes, smoking, drinking, BMI, WBC, TG, TC, LDL-C, HDL-C, or ApoA1 (Table 7).

**Table 7:** Correlation analysis of LncRNA-FA2H-2 and clinical characteristics.

	**LncRNA-FA2H-2**	
	**R**	***P***
Age	0.07	0.44
Gender	−0.80	0.06
Hypertension	0.04	0.38
Diabetes	0.209	0.073
Smoking	0.047	0.19
Drinking	0.05	0.381
BMI	0.43	0.21
WBC	0.128	0.089
TG	0.042	0.66
TC	0.218	0.053
LDL-C	−0.11	0.238
HDL-C	0.05	0.547
ApoA1	−0.07	0.432

### Correlation Analysis Between LncRNA-FA2H-2 and TNF-α, MCP-1, VCAM-1, ICAM-1, and IL-6 in CHD Patients

A negative correlation was found between LncRNA-FA2H-2 and MCP-1, VCAM-1, ICAM-1, IL-6 in CHD patients (Table 8).

**Table 8:** Correlation analysis of LncRNA-FA2H-2 and TNF-α, MCP-1, VCAM-1, ICAM-1, IL-6.

	**LncRNA-FA2H-2**	
	**R**	***P***
TNF-α	0.22	0.82
MCP-1	−0.80	0.04
VCAM-1	−0.09	0.01
ICAM-1	−0.02	0.015
IL-6	−0.22	0.006

### Correlation Analysis Between the Gensini Score and LncRNA-FA2H-2 in CHD Patients

**Figure 3:**
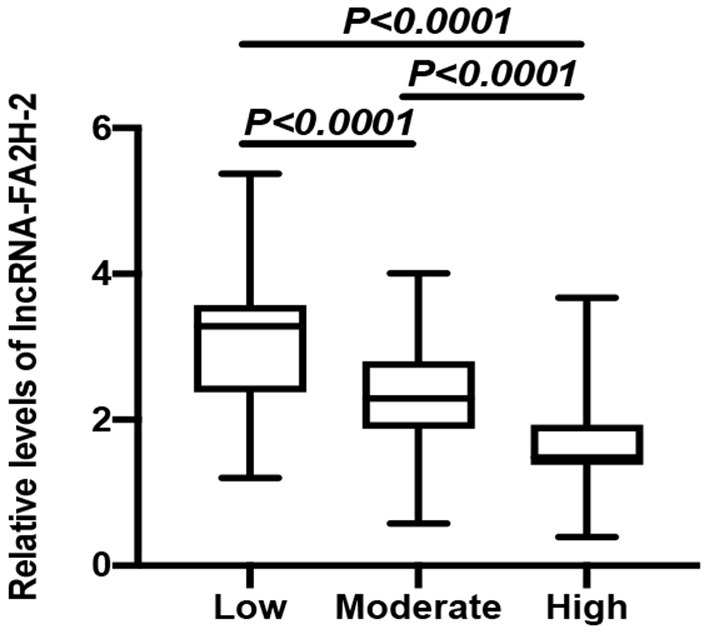
Comparison of relative expressions of LncRNA-FA2H-2 in the high risk, moderate risk, and low risk groups.

CHD patients were grouped into high risk, moderate risk, and low risk groups according to their Gensini score. The data showed that the relative levels of LncRNA-FA2H-2 between the two comparative analyses were statistically significant (Figure 3). A negative correlation was found between the Gensini score and LncRNA-FA2H-2 (Table 9).

**Table 9:** Correlation analysis of the Gensini score and LncRNA-FA2H-2.

	**Gensini score**	
	**R**	***P***
LncRNA-FA2H-2	−0.642	0.032

### Comparison of Sensitivities and Specificities of ROC Curves for TNF-α, MCP-1, VCAM-1, ICAM-1, and IL-6

By performing ROC curves for CHD patients, the cut-off value, sensitivity, and specificity corresponding to the maximum of the Jorden index were calculated. The results revealed that the cut-off value of TNF-α was 290.5, with a sensitivity of 73% and specificity of 64%, and the AUC was 0.832 (0.77, 0.893); the cut-off value of MCP-1 was 396, with a sensitivity of 59% and specificity of 79%, and the AUC was 0.731 (0.653, 0.809); the cut-off value of VCAM- 1 was 264, with a sensitivity of 72% and specificity of 83%, the AUC was 0.822 (0.757, 0.887); the cut-off value of ICAM-1 was 201.5, with a sensitivity of 75% and specificity of 65%, the AUC was 0.794 (0.715, 0.874); the cut-off value of IL-6 was 328, with a sensitivity of 55% and specificity of 90% and the AUC was 0.760 (0.685, 0.834) (Table 10).

**Table 10:** Comparison of the sensitivities and specificities of TNF-α, MCP-1, VCAM-1, ICAM-1, and IL-6.

	**Sensitivity**	**Specificity**	**AUC**	**Yoden index**	**Cut-off**
TNF-α	73%	64%	0.832 (0.77, 0.893)	0.370	290.5
MCP-1	59%	79%	0.731 (0.653, 0.809)	0.380	396
VCAM-1	72%	83%	0.822 (0.757, 0.887)	0.550	264
ICAM-1	75%	65%	0.794 (0.715, 0.874)	0.400	201.5
IL-6	55%	90%	0.760 (0.685, 0.834)	0.450	328

### ROC Curve Analyses of LncRNA-FA2H-2 Sensitivity and Specificity

The ROC curve was performed for CHD patients, and the corresponding critical value, sensitivity, and specificities at the maximum of the Jorden index were calculated. The results indicated that the cut-off value of LncRNA-FA2H-2 was 3.155, with a sensitivity of 85% and specificity of 82%, and the AUC was 0.834 (0.688, 0.85) (Table 11).

**Table 11:** Comparison of the sensitivity and specificity of LncRNA-FA2H-2.

	**Sensitivity**	**Specificity**	**AUC**	**Yoden index**	**Cut-off**
LncRNA-FA2H-2	85%	82%	0.834 (0.688, 0.85)	0.670	3.155

### Binary Logistic Regression Analysis

CHD was used as the dependent variable, inflammation-related factors were included as independent variables, and the values assigned for binary logistic regression analysis showed that TNF-α, MCP-1, VCAM-1, IL-6, and LncRNA-FA2H-2 were independent risk factors for CHD patients (Table 12).

**Table 12:** Binary logistic regression analysis of CHD patients.

	**β**	**Sx**	**Wald**	**RR (95% CI)**	***P***
TNF-α	0.08	0.02	11.549	1.0008 (1.003, 1.013)	0.001
MCP-1	0.06	0.02	10.354	1.006 (1.002, 1.010)	0.001
VCAM-1	0.10	0.03	11.624	1.010 (1.004, 1.015)	0.001
ICAM-1	0.03	0.02	1.185	1.004 (0.998, 1.007)	0.276
IL-6	0.09	0.02	11.797	1.009 (1.004, 1.014)	0.001
LncRNA-FA2H-2	−0.906	0.178	25.939	0.404 (0.285, 0.573)	0.001

## Discussion

The study of pathophysiological changes in CHD patients has shown that inflammatory responses play a key role in the course of CHD. A variety of inflammatory markers are not only present in the walls of sclerotic arteries, but also detectable in the blood, and these inflammatory markers can be used not only for predicting the risk of developing CHD, but also for the evaluation of disease outcomes (24). The selection of inflammatory markers is therefore important for the risk assessment of CHD. Previous studies have shown that some inflammatory markers have important prognostic values for CHD, such as hs-CRP, NLR, IL-8, and IL-10 (25, 26). However, these inflammatory indicators are susceptible to external environmental interference and cannot accurately assess the magnitude of risk for CHD patients. Therefore, the search for reliable and novel inflammatory markers can help to detect cardiovascular disease early and facilitate timely disease monitoring and prognostic assessment.

LncRNAs, which are mainly localized in the cytoplasm or nucleus and do not have a protein-coding function in addition to lacking an obvious open reading frame, have been shown to be key factors in biological processes (21). LncRNAs have been found to be present in whole blood, serum and plasma, breast milk, and urine, and lncRNA was found to remain stable in harsh conditions, such as boiling, extreme pH, and low temperature (27). The stable nature of lncRNAs makes it possible to use them clinically to monitor the pathophysiological status of patients. There is growing evidence that lncRNAs can be used as important biomarkers for risk assessment in cardiovascular disease and may be better indicators among the non-invasive indicators to assess the magnitude of CHD risk. For example, LncRNA ANRIL, and lncRNA H19 are strongly associated with the risk of developing coronary atherosclerosis (28). In addition, RP5-833A20.1 plays a role in promoting atherosclerosis by increasing the expressions of LDL-C and inflammatory factors, and decreasing the expression of HDL-C (29). Studies have confirmed that lncRNA LIPCAR is closely associated with the prognoses of heart failure patients (30). The lncRNA Meg3 is expected to be a new target for the treatment of cardiac remodeling and myocardial fibrosis (31). The lncRNA coronary marker is significantly elevated in the plasma of patients with CHD (32). Another study reported that lncRNA aHIF, ANRIL, and Kcnq1ot1 were closely related with the occurrence of acute myocardial infarction, and that Kcnq1ot1 could be used as a prognostic indicator to determine left ventricular function (33–35).

Using microarray analysis, we previously found that lncRNA-FA2H-2 played an important role in the process of atherosclerosis, and lncRNA-FA2H-2 expression was significantly downregulated and closely associated with inflammatory responses in human atherosclerotic plaque tissues. Our previous study showed that the lesion areas were increased by lncRNA-FA2H-2 knockdown in the aortic root and aortic valve of ApoE^−/−^ mice fed a high fat diet. Moreover, the intima of the aortic root and aortic valve were proliferative and disarranged, and had a large number of lipid-laden foam cells and cholesterol crystals in the lncRNA-FA2H-2 knockdown group. In addition, LncRNA-FA2H-2 knockdown increased VCAM-1, MCP-1, and IL-6 expressions in atherosclerotic lesions in ApoE^−/−^ mice fed a high fat diet. Furthermore, LncRNA-FA2H-2 attenuated the endothelial cell inflammation caused by OX-LDL. Based on the above findings, we propose that lncRNA-FA2H-2 may be used as a potential inflammatory biomarker for CHD. In the current study, we verified the hypothesis by examining the relative expressions of lncRNA-FA2H-2 and inflammatory markers in CHD patients, and the correlation between lncRNA-FA2H-2 and inflammatory responses. We found statistically significant expression levels of TG, HDL-C, ApoA1, and CRP in CHD patients, consistent with the results of previous studies (36). However, we also found that there was no statistically significant difference in diabetes, smoking, and drinking in CHD patients. We speculate that possible reasons for diabetes and smoking not being highly significant were as follows: (1) The enrolled patients were region-specific, only the patients in Henan Province were involved, and all of them were Han Chinese. (2) Due to regional specificities of the enrolled patients, there may have been deviations from large-scale studies that were not representative. Therefore, in the next study, we should increase the sample size and conduct a multi-center study. (3) Patient information was obtained from the hospital's electronic medical record system and lifestyles other than smoking and drinking were not recorded in the system.

To further investigate the role of inflammatory factors in CHD patients, we measured the expression levels of inflammation-related indicators by ELISA and found that the levels of TNF-α, MCP-1, VCAM-1, ICAM-1, and IL-6 were significantly elevated in CHD patients. In addition, the Gensini score represents the degree of coronary lumen stenosis. The results showed that the relative levels of TNF-α, MCP-1, VCAM-1, ICAM-1, and IL-6 were associated with the Gensini score and degree of risk, suggesting that the expression levels of inflammatory markers correlated with the severity of CHD. The reason for these findings may be that the more severe the degree of coronary stenosis, the more intense the inflammatory response. Therefore, it can be tentatively concluded that the occurrence and development of CHD are inextricably linked to the inflammatory response. To further investigate the expression levels of lncRNA-FA2H-2 in patients with CHD, we determined lncRNA-FA2H-2 expression using qRT-PCR and found that its expression levels were significantly decreased in CHD patients, which is consistent with our previous *in vitro* results. More importantly, lncRNA-FA2H-2 negatively correlated with MCP-1, VCAM-1, ICAM-1, and IL-6, further confirming the potential of lncRNA-FA2H-2 as an inflammatory biomarker in CHD patients. Importantly, we found that lncRNA-FA2H-2 was correlated with the Gensini score, suggesting that lncRNA-FA2H-2 was related to the degree of stenosis and severity in CHD. In addition, the specificity of lncRNA-FA2H-2 was lower than that of other inflammatory indexes, which was considered to be due to the enrolled population of patients who underwent coronary angiography, and was different from a true healthy control group. However, the AUC was larger than the other indicators, indicating that the diagnosis had a certain advantage over the rest of the inflammatory indicators, but the specific mechanism was unclear. A possible reason for this is that lncRNA-FA2H-2 is more stable and lncRNA-FA2H-2 may affect the occurrence and development of CHD through other pathways, although, further verification of this possibility is needed. In addition, using logistic analysis we found that lncRNA-FA2H-2 was a risk factor for CHD, suggesting that lncRNA-FA2H-2 is expected to be a predictor for the development of CHD.

The above results were limited by the following factors: (1) This was a single-center study with a small sample size, and the study subjects had differences in clinical baseline information. Therefore, a larger sample size is needed to verify the results. There are also more risk factors for CHD, so our inclusion index was limited. (2) There may have also been operational errors during the study. (3) Although, lncRNAs exist in plasma and a variety of body fluids, the metabolic characteristics of specific lncRNAs need to be identified. In addition, the cellular origin of the detected circulating lncRNAs is difficult to determine, and we cannot yet eliminate the possibility that these lncRNAs are associated with other underlying diseases in patients with cardiovascular diseases. This could affect the specificity and sensitivity of lncRNA use in the diagnostic and prognostic determination of cardiovascular diseases. Although, studies have demonstrated that lncRNAs played an important role in various cardiovascular disease processes such as CHD, heart failure, and hypertension, further, confirmation using larger sample sizes and plaque tissues from CHD patients is needed to determine whether lncRNA-FA2H-2 can be an inflammatory biomarker and therapeutic target for CHD.

## Data Availability Statement

The original contributions presented in the study are included in the article/supplementary material, further inquiries can be directed to the corresponding author/s.

## Ethics Statement

Written informed consent was obtained from the individual(s) for the publication of any potentially identifiable images or data included in this article.

## Author Contributions

FG and GL conceived and supervised the study and revised the manuscript. FG designed experiments and wrote the manuscript. FG and YS performed experiments. FG and BH analyzed the data. All authors read and approved the final manuscript.

## Conflict of Interest

The authors declare that the research was conducted in the absence of any commercial or financial relationships that could be construed as a potential conflict of interest.
