# Corrigendum: Correlation of Long Non-coding RNA LncRNA-FA2H-2 With Inflammatory Markers in the Peripheral Blood of Patients With Coronary Heart Disease

**DOI:** 10.3389/fcvm.2021.799848

**Published:** 2021-11-17

**Authors:** Fengxia Guo, Yanhua Sha, Bing Hu, Gang Li

**Affiliations:** ^1^Department of Clinical Laboratory, Henan Provincial People's Hospital, People's Hospital of Zhengzhou University, Zhengzhou, China; ^2^Department of Laboratory Medicine, The Second Affiliated Hospital of Guangzhou University of Chinese Medicine, Guangzhou, China; ^3^Department of Clinical Laboratory, Affiliated Cancer Hospital of Zhengzhou University, Zhengzhou, China

**Keywords:** coronary heart disease, long non-coding RNA, inflammatory marker, LncRNA-FA2H-2, peripheral blood

**Incorrect Affiliation**

In the published article, there was an error in affiliation 1. Instead of “Department of Clinical Laboratory, Henan Provincial People' Hospital, People' Hospital of Zhengzhou University, Zhengzhou, China”, it should be “Department of Clinical Laboratory, Henan Provincial People's Hospital, People's Hospital of Zhengzhou University, Zhengzhou, China”.

The authors apologize for this error and state that this does not change the scientific conclusions of the article in any way. The original article has been updated.

## Publisher's Note

All claims expressed in this article are solely those of the authors and do not necessarily represent those of their affiliated organizations, or those of the publisher, the editors and the reviewers. Any product that may be evaluated in this article, or claim that may be made by its manufacturer, is not guaranteed or endorsed by the publisher.

